# PK/PD analysis of biapenem in patients undergoing continuous hemodiafiltration

**DOI:** 10.1186/s40780-015-0031-6

**Published:** 2015-11-14

**Authors:** Gaku Akashita, Yuto Hosaka, Toru Noda, Kazuya Isoda, Tsutomu Shimada, Kazuki Sawamoto, Ken-ichi Miyamoto, Takumi Taniguchi, Yoshimichi Sai

**Affiliations:** Department of Medicinal Informatics, Graduate School of Medical Sciences, Kanazawa University, 13-1 Takara-machi, Kanazawa, 920-8640 Japan; Department of Hospital Pharmacy, University Hospital, Kanazawa University, 13-1 Takara-machi, Kanazawa, 920-8641 Japan; School of Pharmacy, College of Medical, Pharmaceutical and Health Sciences, Kanazawa University, Kakuma-machi, Kanazawa, 920-1192 Japan; Intensive Care Unit, University Hospital, Kanazawa University, 13-1 Takara-machi, Kanazawa, 920-8641 Japan

**Keywords:** Biapenem, Continuous hemodiafiltration, Pharmacokinetics, Monte Carlo simulation, PK/PD breakpoint

## Abstract

**Background:**

Continuous hemodiafiltration (CHDF) is used as renal replacement therapy for critically ill patients with renal failure, and to treat hypercytokinemia. Since CHDF also clears therapeutic agents, drug pharmacokinetics (PK) should be dependent upon CHDF conditions. Although the antibiotic biapenem (BIPM) is used in patients undergoing CHDF, the optimal therapeutic regimen in such patients has not been fully clarified. In this study, we investigated the PK of BIPM in patients with various levels of renal function undergoing CHDF with polysulfone (PS) membrane, and used PK models to identify the optimal administration regimen.

**Methods:**

BIPM (300 mg) was administered by infusion in patients undergoing CHDF (*n* = 7). Blood and filtrate-dialysate were collected for compartment and non-compartment analysis.

**Results:**

The sieving coefficient of PS membrane was 1.00 ± 0.06 (mean ± S.D., *n* = 7), and CHDF clearance of BIPM was found to be the sum of the dialysate flow rate (Q_D_) and filtrate flow rate (Q_F_). Non-CHDF clearance showed inter-individual variability (4.82 ± 2.48 L/h), depending on residual renal function and non-renal clearance. Based on the average PK parameters obtained with a compartmental model, maximal kill end point (over 40 % T > MIC_4 μg/mL_) was achieved with regimens of 300 mg every 6 h, 300 mg every 8 h, and 600 mg every 12 h. Monte Carlo simulation indicated that 300 mg infusion for 1 h every 6 h was optimal, and the probability of target attainment at MIC_2 μg/mL_ was 90.2 %.

**Conclusions:**

Our results establish the optimal regimen of BIPM in patients with various levels of renal function undergoing CHDF with a PS membrane.

## Background

Continuous hemodiafiltration (CHDF) is a blood purification method that provides alternative functionality for patients with kidney failure. It removes a wide range of compounds, ranging from low-molecular-weight products such as urea nitrogen and creatinine to medium-molecular-weight compounds such as β-2 microglobulin and cytokines [1, 2]. Therefore, CHDF is widely used in intensive care units to treat patients with unstable hemodynamic pathologies, including acute pancreatitis, fulminant hepatitis, and acute renal failure, as well as to remove cytokines in sepsis patients, even if they have normal renal function [3–6]. Since the pharmacokinetics (PK) of medications may be affected by elimination through dialysis and filtration during CHDF therapy, it is necessary to select an appropriate administration regimen (timing, dose amount, and dose rate) depending upon the conditions of CHDF [7, 8]. It has also been reported that drug clearance varies depending on the material of the dialysis membrane [9], and cytokine clearance is also dependent on the dialysis membrane material [2].

Biapenem (BIPM) is a carbapenem antibacterial agent with broad-spectrum, potent activity against Gram-positive, Gram-negative, and anaerobic bacteria [10]. It is not cleaved by dehydropeptidase-1 (DHP-1), unlike other penem antibiotics such as panipenem and imipenem, and can be used as a single agent without the need for formulation of a nephrotoxicity-reducing agent or DHP-1 inhibitor [11–13]. Carbapenems are classified as time-dependent antibiotics, and the clinical outcome is closely correlated with the duration for which the drug concentration remains at or above the minimum inhibitory concentration (MIC); the time above the MIC (T > MIC) needs to be over 20 and 40 % of the dosing interval to achieve stasis and maximal kill as end points, respectively [14, 15]. For carbapenem antibiotics, sensitive MIC and intermediate MIC are defined as ≥ 4 μg/mL and ≥ 8 μg/mL, respectively [16]. To obtain optimum effects of BIPM, we need to predict T > MIC from the PK parameters of BIPM. BIPM is mainly excreted from the kidneys, and clearance of BIPM in patients with renal failure is decreased to 2.62 L/h, i.e., approximately 20 % of that of healthy adults [17]. CHDF also alters the clearance of BIPM, and Ikawa et al. have reported on PK modeling and dosage adaptation of BIPM during CHDF with a polymethyl methacrylate (PMMA) membrane in patients with renal failure [18]. The PK of single BIPM administration was calculated using multi-compartment models, and it was found that the dose amount and dosing interval were important factors determining the value of % of T > MIC. However, their models were applicable only to patients with renal failure. Since CHDF is also used for patients with various levels of renal function, models that take account of this are required. Furthermore, the effects of other types of filter membrane in CHDF should also be considered.

In the present study, we developed non-compartmental and compartmental models for repeated BIPM treatment in patients with various levels of renal function undergoing CHDF with a polysulfone (PS) membrane, and examined the optimum treatment regimen by means of Monte Carlo simulation.

## Methods

### Patients

This study was approved by the Clinical Research Ethics Committee of Kanazawa University Hospital (2011–052). Adult patients older than 20 years who were receiving CHDF for acute renal failure or removal of cytokines, and who were prescribed BIPM to treat infection were eligible. Prior to the start of the investigation, informed consent was obtained in writing from each participant or his/her relatives. We excluded patients taking sodium valproate, or with a history of hypersensitivity to carbapenems or fourth-generation cephems, or with contraindications for carbapenems or fourth-generation cephems, or with a history of epilepsy or central nervous system damage. Seven patients (5 males, 2 females) were enrolled. Demographic and medical characteristics of each patient are summarized in Table 1. The glomerular filtration rate (GFR) of each patient was evaluated before CHDF application.

**Table 1:** Patient characteristics

Patient	Sex	Age (years)	Ht (cm)	BW (kg)	BSA (m^2^)	Infusion Interval (hr)	Dose (mg)	Infusion time (hr)	GFR (mL/min/body)
A	M	64	167	95.1	2.03	12	300	1	20.4
B	M	70	157	53.1	1.50	8	300	1	17.0
C	F	33	160	57.5	1.61	12	300	1	15.8
D	M	65	166	65.0	1.70	6	300	1	58.8
E	M	75	162	47.8	1.45	8	300	1	31.0
F	M	55	164	64.8	1.69	6	300	0.5	63.3
G	F	80	145	66.7	1.60	8	300	0.5	8.4
Mean		63.1	160.1	64.3	1.65				30.7
SD		15.6	7.5	15.3	0.19				21.9

*Ht* height; *BW* body weight; *BSA* body surface area; *GFR* glomerular filtration rate (value before CHDF); *SD* standard deviation

### CHDF

In the present study, a PS hemofilter with a membrane surface area of 1.3 m^2^ (EXCELFLO® AEF-13, Asahi Kasei Medical Co., Japan) was used for hemopurification. CHDF was performed basically with a blood flow rate (Q_B_) of 80 mL/min, dialysate flow rate (Q_D_) of 500 mL/h, substitution flow rate (Q_S_) of 500 mL/h, and filtration flow rate (Q_F_) of 1000 mL/h, and was regulated appropriately based on each patient’s status. Sublood-BS® (Fuso Pharmacy, Inc., Japan) was used as a dialysate and also served as a substitution fluid for post-dilutional infusion. During CHDF, nafamostat mesylate was serially administered at a rate of approximately 20 mg/h to avoid coagulation within the circuit.

### BIPM administration and collection of blood and filtrate-dialysate samples

Three hundred milligrams of BIPM (Omegacin® 0.3 g, Meiji Seika Pharma Co., Japan) was administered by intravenous drip infusion for 0.5 or 1 h at 6, 8, or 12 h intervals (Table 1). Blood samples were taken from the blood access port in the extracorporeal circuit proximal to the filter before dosing, at 0, 0.5, 1, 2, and 4 h after infusion, and just before the next infusion. Filtrate-dialysate (FD) samples were simultaneously collected from the filtrate tube to determine the sieving coefficient (SC) and CHDF clearance. After collection, each blood sample was centrifuged immediately. The plasma and supernatants of FD samples were immediately frozen at −80 °C and stored until assay.

### Method of quantitative analysis of BIPM

Quantitative analysis of BIPM in plasma and FD was performed using high-performance liquid chromatography (HPLC) [18]. Briefly, plasma or FD sample (200 μL) was mixed with 200 μL of 1 M 3-morpholinopropane-1-sulfonic acid buffer. An aliquot (300 μL) was transferred to an ultrafiltration device (Nanosep® Centrifugal Devices 10 K, Pall Life Sciences, USA), and centrifuged at 15,000 g for 15 min at 15 °C. An aliquot of the ultrafiltered solution (20 μL) was injected into an HPLC system (Prominence, Shimadzu Co., Japan) equipped with an Octadecyl silica column (Shim-pack CLC-ODS 5 μm (150 x 6 i.d.), Shimadzu Co., Japan) and a UV detector (232 nm). The mobile phase of 1.5 % acetonitrile and 98.5 % 0.1 M sodium acetate buffer (pH 4.6) was delivered isocratically at a flow rate of 1.0 mL/min. The auto-sampler temperature was set at 4 °C, and the column temperature at 40 °C. The calibration curve was linear from 0.1 to 50 μg/mL, and the lower limit of detection was 0.1 μg/mL.

### Calculation of PK parameters of BIPM

**Fig. 1:**
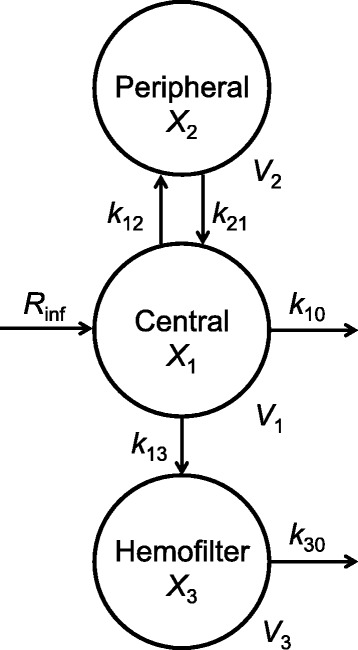
Multicompartment model for BIPM during CHDF. *X*_1_, *X*_2_ and *X*_3_ are the amounts of drug in the central, peripheral and hemofilter compartments, respectively; *V*_1_, *V*_2_ and *V*_3_ are the volumes of the central compartment, peripheral compartment and hemofilter cartridge, respectively; *R*_inf_, drug infusion rate; *k*_10, 30_, elimination rate constants; *k*_12, 13, 21_, rate constants connecting the compartments

Non-compartmental and compartmental analyses were performed using PK analysis software: Numeric Analysis Program for Pharmacokinetics (Napp) ver. 2.31 (Department of Pharmacy, the University of Tokyo Hospital). The area under the BIPM concentration-time curve (AUC) was calculated based on the logarithmic trapezoidal rule. The SC was determined as AUC_FD_/AUC_plasma_. The AUC after first administration was calculated from 0 h to infinity, and subsequent AUCs were estimated from 0 h to the start of the next infusion interval, since the half-life of BIPM in healthy persons is 1 h [19]. The differential equations for mass balances were as follows (Fig. 1):

$$ \frac{d{X}_1}{dt}={R}_{inf} - \left({k}_{10}+{k}_{12}+{k}_{13}\right)\cdot {X}_1 + {k}_{21}\cdot {X}_2 $$

$$ \frac{d{X}_2}{dt}={k}_{12}\cdot {X}_1-{k}_{21}\cdot {X}_2 $$

$$ \frac{d{X}_3}{dt}={k}_{13}\cdot {X}_1-{k}_{30}\cdot {X}_3 $$

where *X*_1_, *X*_2_ and *X*_3_ are the amounts of drug (mg) in the central, peripheral and hemofilter compartment, respectively; *R*_inf_ is the drug infusion rate (mg/hr); *k*_10_ and *k*_30_ are the elimination rate constant (hr^−1^) from the central compartment and the hemofilter compartment, respectively; *k*_12_ and *k*_21_ are the intercompartmental transfer rate constants (hr^−1^); *k*_13_ is transfer rate constant (hr^−1^) from the central compartment to the hemofilter compartment.

These equations were used to obtain the following formulas for the drug concentrations (μg/mL) in the central compartment (C_1_) and FD (C_3_) at time t (hr) during and after administration in the steady state:

(i) 0 ≦ t ≦ T_inf_

$$ {C}_1=\frac{R_{inf}}{V_1}\left\{\frac{\left({k}_{21}-\alpha \right)}{\alpha \left(\beta -\alpha \right)}\left({e}^{\alpha \cdot {T}_{inf}}-1\right)\left(\frac{e^{-\alpha \cdot \tau }}{1-{e}^{-\alpha \cdot \tau }}\right){e}^{-\alpha \cdotp t}+\frac{\left({k}_{21}-\beta \right)}{\beta \left(\alpha -\beta \right)}\left({e}^{\beta \cdot {T}_{inf}}-1\right)\left(\frac{e^{-\beta \cdot \tau }}{1-{e}^{-\beta \cdot \tau }}\right){e}^{-\beta \cdotp t}+\frac{\left({k}_{21}-\alpha \right)}{\alpha \left(\beta -\alpha \right)}\left(1-{e}^{-\alpha \cdotp t}\right)+\frac{\left({k}_{21}-\beta \right)}{\beta \left(\alpha -\beta \right)}\left(1-{e}^{-\beta \cdotp t}\right)\right\} $$

$$ {C}_3=\frac{R_{inf}\cdotp {k}_{13}}{V_3}\left\{\frac{\left({k}_{21}-\alpha \right)}{\alpha \left(\beta -\alpha \right)\left({k}_{30}-\alpha \right)}\left({e}^{\alpha \cdotp {T}_{inf}}-1\right)\left(\frac{e^{-\alpha \cdotp \tau }}{1-{e}^{-\alpha \cdotp \tau }}\right){e}^{-\alpha \cdotp t}+\frac{\left({k}_{21}-\beta \right)}{\beta \left(\alpha -\beta \right)\left({k}_{30}-\beta \right)}\left({e}^{\beta \cdotp {T}_{inf}}-1\right)\left(\frac{e^{-\beta \cdotp \tau }}{1-{e}^{-\beta \cdotp \tau }}\right){e}^{-\beta \cdotp t}+\frac{\left({k}_{21}-{k}_{30}\right)}{k_{30}\left(\alpha -{k}_{30}\right)\left(\beta -{k}_{30}\right)}\left({e}^{k_{30}\cdotp {T}_{inf}}-1\right)\left(\frac{e^{-{k}_{30}\cdotp \tau }}{1-{e}^{-{k}_{30}\cdotp \tau }}\right){e}^{-{k}_{30}\cdotp t}+\frac{\left({k}_{21}-\alpha \right)}{\alpha \left(\beta -\alpha \right)\left({k}_{30}-\alpha \right)}\left(1-{e}^{-\alpha \cdotp t}\right)+\frac{\left({k}_{21}-\beta \right)}{\beta \left(\alpha -\beta \right)\left({k}_{30}-\beta \right)}\left(1-{e}^{-\beta \cdotp t}\right)+\frac{\left({k}_{21}-{k}_{30}\right)}{k_{30}\left(\alpha -{k}_{30}\right)\left(\beta -{k}_{30}\right)}\left(1-{e}^{-{k}_{30}\cdotp t}\right)\right\} $$

(ii) t > T_inf_

$$ {C}_1=\frac{R_{inf}}{V_1}\left\{\frac{\left({k}_{21}-\alpha \right)}{\alpha \left(\beta -\alpha \right)}\left({e}^{\alpha \cdotp {T}_{inf}}-1\right)\left(\frac{1}{1-{e}^{-\alpha \cdotp \tau }}\right){e}^{-\alpha \cdotp t}+\frac{\left({k}_{21}-\beta \right)}{\beta \left(\alpha -\beta \right)}\left({e}^{\beta \cdotp {T}_{inf}}-1\right)\left(\frac{1}{1-{e}^{-\beta \cdotp \tau }}\right){e}^{-\beta \cdotp t}\right\} $$

$$ {C}_3=\frac{R_{inf}\cdotp {k}_{13}}{V_3}\left\{\frac{\left({k}_{21}-\alpha \right)}{\alpha \left(\beta -\alpha \right)\left({k}_{30}-\alpha \right)}\left({e}^{\alpha \cdotp {T}_{inf}}-1\right)\left(\frac{1}{1-{e}^{-\alpha \cdotp \tau }}\right){e}^{-\alpha \cdotp t}+\frac{\left({k}_{21}-\beta \right)}{\beta \left(\alpha -\beta \right)\left({k}_{30}-\beta \right)}\left({e}^{\beta \cdotp {T}_{inf}}-1\right)\left(\frac{1}{1-{e}^{-\beta \cdotp \tau }}\right){e}^{-\beta \cdotp t}+\frac{\left({k}_{21}-{k}_{30}\right)}{k_{30}\left(\alpha -{k}_{30}\right)\left(\beta -{k}_{30}\right)}\left({e}^{k_{30}\cdotp {T}_{inf}}-1\right)\left(\frac{1}{1-{e}^{-{k}_{30}\cdotp \tau }}\right){e}^{-{k}_{30}\cdotp t}\right\} $$

Where T_inf_ is the drug infusion time (hr); τ is the inter-dose interval (hr); *V*_1_ is the volume of distribution of the central compartment; *V*_3_ is the blood volume of the hemofilter cartridge; and α and β are macro rate constants (hr^−1^) expressed as α + β = *k*_12_ + *k*_21_ + *k*_10_ + *k*_13_, αβ = *k*_21_ · *k*_10_ + *k*_21_ · *k*_13_.

The rate constants *k*_13_ and *k*_30_ can be expressed as:

$$ {k}_{13}=\frac{\left({Q}_F+{Q}_D\right)\cdotp SC}{V_1} $$

$$ {k}_{30}=\frac{\left({Q}_F+{Q}_D\right)}{V_3} $$

Where Q_F_ is the filtrate flow rate (L/hr); Q_D_ is the dialysate flow rate (L/hr).

The clearance in CHDF (CL_CHDF_) was calculated as (Q_F_ + Q_D_) · SC and the clearance by non-CHDF routes (CL_non-CHDF_) was estimated as *k*_10_ · *V*_1_.

We modified the formula of Ikawa et al. [18] to include data obtained not only after the first administration of BIPM, but also those obtained during repeated administration of BIPM. The concentration-time data of BIPM in plasma and FD were concurrently fitted to the multi-compartment model described above.

Glomerular filtration rate (GFR) (mL/min/body) was calculated as follows:

$$ GFR=\frac{eGFR\cdot BSA}{1.73} $$

$$ male\ :\  eGFR=194\cdot Sc{r}^{-1.094}\cdot Ag{e}^{-0.287} $$

$$ BSA=\frac{105.29\cdot H{t}^{0.619}\cdot B{W}^{0.460}}{10,000} $$

$$ female\ : eGFR=194\cdot Sc{r}^{-1.094}\cdot Ag{e}^{-0.287}\cdot 0.739 $$

$$ BSA=\frac{82.84\cdot H{t}^{0.689}\cdot B{W}^{0.437}}{10,000} $$

Where eGFR is estimated glomerular filtration rate (mL/min/1.73 m^2^); BSA is body surface area (m^2^) [20]; Scr is serum creatinine concentration (mg/dL); Age is given in years; Ht is height (cm); BW is body weight (kg). eGFR was determined just before CHDF application.

### Calculation of the PK/PD breakpoint by Monte Carlo simulation

Monte Carlo simulation was performed with the normal random number generation function of Microsoft® Excel® 2007 (Microsoft, USA). We generated a population parameter set of 10,000 cases using a mean and variance of *k*_21_, *V*_1_, α and β obtained by the standard two-stage method (compartmental analysis), thereby generating the BIPM plasma concentration transition of 10,000 cases. After setting the dosing interval and administration time of BIPM in the above formula (C_1_), the exposure time for which the BIPM plasma concentration remained at the MIC was finally calculated as the cumulative percentage over a 24 h period in the BIPM plasma concentration transition of the 10,000 cases [21–23]. Then, we calculated the number of cases that showed T > MIC of 40 % or more at each PK/PD target value in each regimen. Maximum MIC of more than 80 % was set as the PK/PD breakpoint probability of target attainment (PTA) in each regimen.

## Results

In the present study, no adverse events or laboratory abnormalities were noted that were definitely attributable to BIPM.

**Fig. 2:**
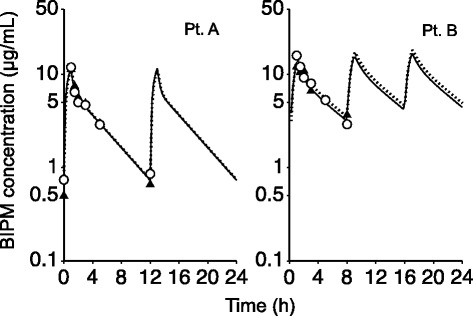
Typical fittings between the plasma and FD concentration simulation curves and the observed values. Simulation curves of plasma (open circles, solid line) and FD (closed triangle, dotted line) concentration were obtained by compartmental analysis

**Fig. 3:**
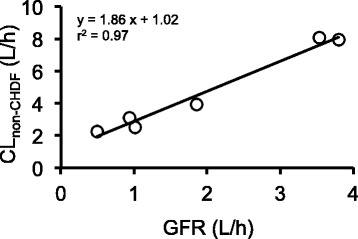
Correlation between individual GFR and CL_non-CHDF_. The solid line indicates the linear regression line

SC of the PS membrane was calculated to be 1.00 ± 0.06 by non-compartmental analysis (Table 2). The PK parameters estimated by compartmental analysis were as follows: *V*_1_, 13.46 ± 5.29 L; *k*_12_, 0.75 ± 0.71 h^−1^; *k*_21_, 0.95 ± 0.27 h^−1^; *k*_10_, 0.39 ± 0.22 h^−1^; and *k*_13_, 0.13 ± 0.04 h^−1^ (Table 3). Figure 2 shows typical fittings between the plasma and FD concentration time curves obtained by compartmental analysis and the real values in Pt. A and Pt. B. Time courses of BIPM concentration in plasma and FD obtained by compartmental analysis in other patients also closely fitted the observed values. CL_CHDF_ and CL_non-CHDF_ were estimated to be 1.53 ± 0.10 L/h and 4.86 ± 2.50 L/h, respectively. The inter-individual variation in CL_non-CHDF_ was large. The sum of CL_CDHF_and CL_non-CHDF_, calculated as (Q_F_ + Q_D_) · SC and as *k*_10_ · *V*_1_, respectively [x], and CL_tot_ obtained by non-compartmental analysis of the plasma BIPM concentration [y] showed a good correlation (*y* = 1.01 x − 0.02, *r*^*2*^ = 1.00), supporting the validity of the model. As shown in Fig. 3, the correlation between GFR obtained just before CHDF and CL_non-CHDF_ was also high (*r*^*2*^ = 0.97), and the slope and y-intercept were 1.86 and 1.02, respectively. In this study, the number of patients who showed T > MIC_4 μg/mL_ values of 40 % or more was 6, and one was in the range of 20 to 40 %. Moreover, the number of patients with T > MIC_8 μg/mL_ values of more than 40 % was one, and 6 were in the range of 20 to 40 %.

**Table 2:** Pharmacokinetic parameters calculated by non-compartmental analysis

Patient	AUC_0→__τ__, plasma_(mg・hr/L)	AUC_0→__τ__, FD_(mg・hr/L)	SC	CL_tot_(L/hr)	Vd_ss_(L)	C_max, plasma_(μg/mL)	C_max, FD_(μg/mL)	T_1/2,__plasma_(hr)	T_1/2,__FD_(hr)
A	37.7	37.6	1.00	7.96	0.33	11.92	11.29	3.99	3.20
B	72.4^a^	79.7^a^	1.10	4.14	0.46	15.93	12.09	3.97	5.43
C	63.2	61.2	0.97	4.75	0.30	20.08	19.43	3.42	3.62
D	31.4	29.9	0.95	9.54	0.35	12.15	9.13	2.05	2.20
E	54.3	55.3	1.02	5.53	0.33	19.42	19.55	2.83	2.73
F	32.2	29.9	0.93	9.32	0.24	23.93	15.37	1.38	1.25
G	78.2	79.6	1.02	3.84	0.17	25.80	24.24	3.39	3.66
Mean			1.00	6.44	0.31			3.00	3.15
SD			0.06	2.45	0.09			0.99	1.31

*AUC*_*0→*__*τ*__*, plasma*_ area under the plasma concentration-time curve from time 0 to next infusion; *AUC*_*0→*__*τ*__*, FD*_ area under the filtrate-dialysate concentration-time curve from time 0 to next infusion; *SC* sieving coefficient (AUC_0→__τ__, FD_/AUC_0→__τ__, Plasma_); *CL*_*tot*_ total clearance (300/AUC_0→_τ, plasma); *Vd*_*ss*_ volume of distribution at the steady state; *C*_*max,plasma*_ maximum concentration of BIPM in plasma; *C*_*max, FD*_ maximum concentration of BIPM in filtrate-dialysate; *T*_*1/2, plasma*_ elimination half-life of BIPM in plasma; *T*_*1/2, FD*_ elimination half-life of BIPM in filtrate-dialysate; *SD* standard deviation

^a^AUC_0→∞_

**Table 3:** Pharmacokinetic parameters of biapenem after intravenous administration of biapenem (300 mg) during CHDF

Patient	*V*_1_ (L)	*k*_12_ (hr^−1^)	*k*_21_ (hr^−1^)	*k*_10_ (hr^−1^)	*k*_13_ (hr^−1^)	CL_CHDF_ (L/hr)	CL_non-CHDF_ (L/hr)
A	11.09	2.26	1.10	0.56	0.14	1.53	6.24
B	17.45	0.33	0.66	0.15	0.09	1.65	2.54
C	9.51	0.74	0.92	0.33	0.16	1.55	3.11
D	23.84	0.18	1.13	0.34	0.06	1.43	8.04
E	11.74	0.26	0.51	0.33	0.14	1.63	3.93
F	10.19	0.69	1.08	0.78	0.14	1.39	7.92
G	10.43	0.79	1.25	0.22	0.15	1.53	2.27
Mean	13.46	0.75	0.95	0.39	0.13	1.53	4.86
SD	5.29	0.71	0.27	0.22	0.04	0.10	2.50

*V*_*1*_ Volume of distribution of central compartment; *k*_12_ transfer rate constant from central compartment to peripheral compartment; *k*_21_ transfer rate constant from peripheral compartment to central compartment; *k*_10_ elimination rate constant from central compartment; *k*_13_ transfer rate constant from central compartment to filtrate-dialysate side compartment (Q_F_ + Q_D_) · SC/*V*_1_; *CL*_*CHDF*_ clearance by CHDF (Q_F_ + Q_D_) · SC; *CL*_*non-CHDF*_ clearance by non-CHDF routes (*k*_10_ · *V*_1_); *SD* standard deviation

**Fig. 4:**
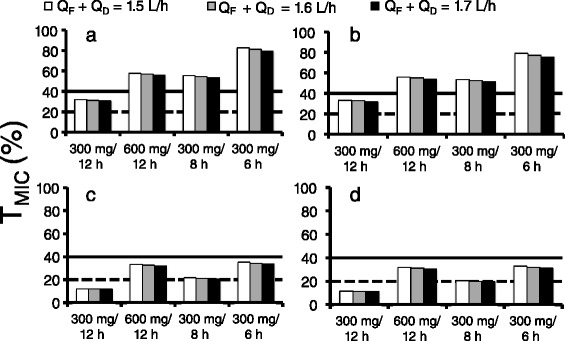
Values of T > MIC of 4 μg/mL and 8 μg/mL in different regimens. Panel **a** represents the case of MIC = 4 μg/mL and infusion time 1 h; Panel **b** represents the case of MIC = 4 μg/mL and infusion time 0.5 h; Panel **c** represents the case of MIC = 8 μg/mL and infusion time 1 h; Panel **d** represents the case of MIC = 8 μg/mL and infusion time 0.5 h. The solid line represents 40 % T > MIC. The dotted line represents 20 % T > MIC

Figure 4 shows % of T > MIC values in plasma of patients undergoing CHDF based on the average parameters of the multi-compartment models on various regimens, including different values of infusion time, MIC values, sum of Q_F_ and Q_D_, dosage and administration. As Q_F_ + Q_D_ increased, % of T > MIC slightly decreased. In addition, % of T > MIC for 1 h infusion was higher than that for 0.5 h infusion under the same conditions of Q_F_ + Q_D_ and administration dose. The most effective regimen was 300 mg every 6 h given by 1 h infusion, with a Q_F_ + Q_D_ value of 1.5 L/h. This method could achieve % of T > MIC of 82.8 % at MIC = 4 μg/mL, and 35.1 % at MIC = 8 μg/mL.

**Fig. 5:**
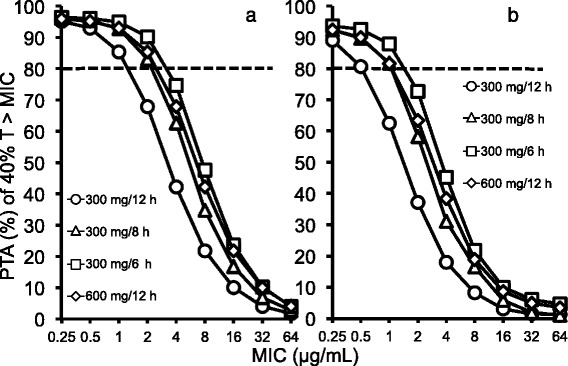
PTA of 40 % T > MIC in plasma at specific MICs for different BIPM regimens. The dotted line represents 80 % PTA. Panel **a**: infusion time of 1 h; Panel **b**: infusion time of 0.5 h

Based on Monte Carlo simulation of the case of 0.5 h infusion, the PK/PD breakpoint was 0.5 μg/mL in the regimen of dosage 300 mg every 12 h, and was 1.0 μg/mL in the regimens of dosage 300 mg every 8 h, dosage 300 mg every 6 h, and dosage 600 mg every 12 h (Fig. 5). In the case of 1 h infusion, the PK/PD breakpoint was 1.0 μg/mL in the regimen of dosage 300 mg every 12 h, and 2.0 μg/mL in the regimens of dosage 300 mg every 8 h, dosage 300 mg every 6 h, and dosage 600 mg every 12 h. The PTAs were greater for 1 h infusion than for 0.5 h infusion in all cases. Among them, PTA of the regimen of dosage 300 mg every 6 h showed the highest value (90.2 %). The Japan Society of Chemotherapy defines the clinical breakpoint as more than 80 %, though more than 90 % of the PTA has become a reference value of the PK/PD breakpoint outside of Japan.

## Discussion

In this study, we investigated the PK of BIPM using compartmental and non-compartmental analysis in patients undergoing CHDF who had various levels of renal function, and examined the suitability of various administration regimens in terms of PK/PD breakpoint for various bacteria, by means of Monte Carlo simulation.

Ikawa et al. analyzed time courses of BIPM concentration in plasma and FD after single administration in renal failure patients undergoing CHDF [18]. Here, we aimed to obtain a model to analyze the results of repeated administration of BIPM in patients undergoing CHDF who retained various levels of renal function. Time courses of BIPM concentration in plasma and FD in patients undergoing CHDF closely fitted the observed values, suggesting that the constructed model formula successfully represents the results of repeated administration of BIPM in patients undergoing CHDF. In the present study, SC (indicating penetration from blood to dialyzer) was almost 1.0 for the PS membrane material, which is almost the same as the value for PMMA membrane [18, 24]. Thus, there appears to be essentially no difference between PS and PMMA membranes in regard to drug penetration [18]. These results seem consistent with the characteristics of BIPM, such as low molecular weight and low protein binding rate.

We found a strong correlation between the sum of CL_non-CHDF_ and CL_CHDF_ obtained by compartmental analysis and total clearance obtained by non-compartmental analysis (*r*^*2*^ = 1.00), indicating that the compartment model formula was appropriate. Ikawa et al. reported that CL_CHDF_ (1.29 ± 0.08 L/h) was almost the same as the sum of Q_F_ and Q_D_ (1.4 L/h) in CHDF with a PMMA membrane [18]. Suyama et al. also reported that CL_CHDF_ (1.28 ± 0.14 L/h) was almost the same as the sum of Q_F_ and Q_D_ (1.4 L/h) in CHDF with a PMMA membrane [24]. In this study, CL_CHDF_ (1.5 ± 0.1 L/h) estimated by compartmental analysis was similar to the sum of Q_F_ and Q_D_ (1.5–1.7 L/h) in CHDF with a PS membrane. Considering the SC estimated by non-compartmental analysis, CL_CHDF_ of BIPM would be determined by the sum of dialysate flow rate and filtration flow rate. On the other hand, CL_non-CHDF_ represents the sum of non-renal clearance and residual renal clearance. Metabolism of BIPM in kidney and other tissues would contribute to CL_non-CHDF_. Nakajima et al. detected two DHP-1 metabolites of BIPM, L-cysteine and L-cystine, in urine, and reported that excretion of urinary metabolites accounted for approximately 15 % of total clearance; these metabolites were not detected in plasma, suggesting that metabolism of BIPM in kidney contributes to residual renal clearance [19]. DHP-1 is also expressed in the ascending colon and ileum as well as the kidney, and the activity of DHP-1 in ileum has been reported to be twice that in kidney, suggesting that non-renal DHP-1 function in ileum would also contribute to non-renal clearance [25]. The relationship between GFR calculated before CHDF application and CL_non-CHDF_ was given by *y* = 1.86 x + 1.02 (*r*^*2*^ = 0.97) (Fig. 3), and the y-intercept (1.02 L/h) represents non-renal clearance. Non-renal clearance in healthy adults can be estimated as approximately 3.23 L/h, using reported values of total clearance of BIPM in healthy adults of 12.9 ± 1.2 L/h, and urinary excretion rates of unchanged and changed BIPM of approximately 60 and 15 %, respectively [19]. Nagashima et al. reported that non-renal clearance was 2.60 ± 1.55 L/h in patients with renal failure during dialysis with a PS membrane, while CL_tot_ in patients was 2.62 ± 0.60 L/h without HD, so that CL_tot_ was the same as non-renal clearance in patients with renal failure [17]. Non-renal clearance in the present study was a half to one-third of those values. After extracting patients with sepsis from Fig. 3, the y-intercept took a negative value (data not shown), indicating that non-renal clearance would be almost zero in patients with sepsis. In sepsis, hepatic clearance and renal clearance would be diminished owing to the reduced function of systemic organs and impaired blood flow [26], leading to loss of DHP-1 activity in the kidney and other organs. Thus, we consider that the reason why average non-renal clearance in this study was lower than in previous studies is the low values in patients with sepsis.

We investigated the variation in % of T > MIC based on the average values of the PK parameters obtained by compartmental analysis for different regimens, including dose, dosing interval, infusion time, and CHDF conditions applied in our hospital. The % of T > MIC_4 μg/mL_ was more than 40 % in all regimens of 900 mg or more daily dose, regardless of the CHDF conditions and infusion time. Ikawa et al. also reported that although the regimen of 300 mg every 12 h failed to achieve T > MIC_4 μg/mL_ of more than 40 %, the regimen of 600 mg every 12 h did do so [18]. These results indicate that the minimum dosage regimen required to achieve T > MIC_4 μg/mL_ of more than 40 % is 300 mg every 8 h (total amount: 900 mg). On the other hand, no regimen gave % of T > MIC_8 μg/mL_ of more than 40 %, suggesting that these regimens could not achieve the maximal kill end point. However, since the % of T > MIC values obtained using average PK parameters do not reflect variations of CL_non-CHDF_ associated with partial kidney function, these values may not be adequate as a clinical indicator.

Monte Carlo simulation is a computer modeling process that incorporates variability in pharmacokinetic parameters and the natural MIC distribution within a bacterial population. It can be used to develop interpretive susceptibility criteria based on PK/PD breakpoints [27]. In this study, Monte Carlo simulations with 10,000 cases were performed to examine PK/PD breakpoint using the mean and variance of the PK parameters. As regards infusion time, the PK/PD breakpoint obtained for all regimens with 1 h infusion was higher than that for 0.5 h infusion, suggesting that the 1 h infusion provides a better outcome. In this study, the highest PK/PD breakpoint (over 80 % PTA) was 2 μg/mL with 300 mg every 8 h, 300 mg every 6 h, and 600 mg every 12 h, indicating that these regimens provide a sufficient bactericidal effect in the case of bacteria with MIC_2 μg/mL_, but not MIC_4 μg/mL_. In order to obtain an antimicrobial effect of BIPM towards high MIC bacteria (more than 4 μg/mL), such as *Pseudomonas aeruginosa*, higher dose administration and an appropriate regimen would be needed, although the maximum permitted dose of BIPM in Japan has been set at 1,200 mg/day. Among the regimens examined in this study, 300 mg every 6 h with infusion for 1 h was the optimal regimen, and PTA at MIC_2 μg/mL_ was 90.2 %. Although maximum MIC more than 80 % is adopted as the PK/PD breakpoint in Japan, most other countries define it as more than 90 % [27], so the regimen of 300 mg every 6 h with infusion for 1 h also meets the international standard for MIC_2 μg/mL_ bacteria. Although the PK/PD breakpoint is expected to become a decision criterion for individualized and optimized antibacterial therapy, it is focused primarily on the antibacterial agent. In the future, it will be important to take bacterial character into account as well, for example, by means of antimicrobial susceptibility testing and identification of the causative bacteria of infectious diseases.

The present study has several limitations. Firstly, we performed the Monte Carlo simulation using the mean and variance of the PK parameters obtained with the standard two-stage method because of the small number of cases (total, 7 cases), and this might have resulted in overestimation of the inter-individual variation compared to population pharmacokinetics analysis, such as nonlinear mixed effects modelling (NONMEM®). Further, we did not include a parameter of renal function (e.g. GFR or creatinine clearance) in the Monte Carlo simulation, even though BIPM clearance is correlated with renal function. Therefore, as a next step, it would be desirable to identify the PK/PD breakpoint more precisely in patients undergoing CHDF by means of Monte Carlo simulation of BIPM using population pharmacokinetic parameters including renal function (e.g. GFR and creatinine clearance), based on larger numbers of patients with various levels of renal function. In addition, from the viewpoint of clinical applicability, we did not establish that the optimal regimen (300 mg every 6 h with infusion for 1 h) determined by Monte Carlo simulation is safe, because only one patient received the optimal regimen in this study. Therefore, it is impossible to evaluate the safety of optimal regimen. However, the simulated maximum steady-state plasma concentration with the optimal regimen in the present study was approximately 11–24 μg/mL. This concentration is lower than the maximum steady-state plasma concentration after administration of 600 mg by 1 h infusion every 12 h in healthy adults (32.4 ± 2.32 μg/mL) [19]. Therefore, it seems likely that the optimal regimen would be safe even in renal dysfunction patients undergoing CHDF. Nevertheless, it will be important to confirm the safety of the optimal regimen, and clinicians should carefully consider the appropriate regimen when administering BIPM to renal dysfunction patients undergoing CHDF, in addition to monitoring for side effects.

## Conclusions

In the present study, we used PK modeling to establish the optimal regimen of BIPM in patients with various levels of renal function undergoing CHDF. Clearance of CHDF was determined by the flow rate of dialysate and filtration conditions, since almost BIPM flowed into the dialyzer. The results of Monte Carlo simulation indicated that the regimen of 300 mg every 6 h infusion for 1 h was optimal, compared to other regimens used in our hospital. This regimen showed effective antibacterial activity towards MIC_2 μg/mL_ bacteria.

## Abbreviations

CHDF: Continuous hemodiafiltration
PK: Pharmacokinetics
BIPM: Biapenem
DHP-1: Dehydropeptidase-1
MIC: Minimum inhibitory concentration
T > MIC: Time above the MIC
PMMA: Polymethyl methacrylate
PS: Polysulfone
GFR: Glomerular filtration rate
Q_B_: Blood flow rate
Q_D_: Dialysate flow rate
Q_S_: Substitution flow rate
Q_F_: Filtration flow rate
FD: Filtrate-dialysate
SC: Sieving coefficient
HPLC: High-performance liquid chromatography
Napp: Numeric Analysis Program for Pharmacokinetics
AUC: Area under the concentration-time curve
PTA: Probability of target attainment
